# Supplementary figures and images for: Application of phage display technology for the production of antibodies against Streptococcus suis serotype 2
Source: PLoS One. 2021 Oct 26;16(10):e0258931. doi: 10.1371/journal.pone.0258931 (PMC8547629; doi:10.1371/journal.pone.0258931)

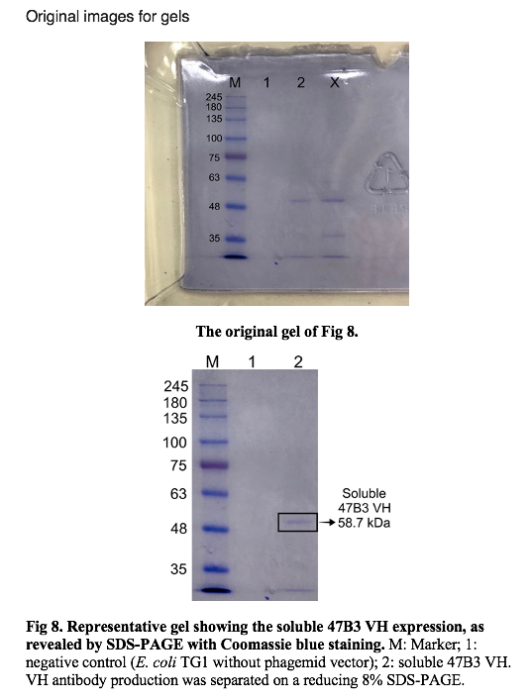

Supplement: S1 Raw images — (TIFF) [file pone.0258931.s001.tiff]
